# Early hybrid cardiac rehabilitation in congenital heart disease: the QUALIREHAB trial

**DOI:** 10.1093/eurheartj/ehae085

**Published:** 2024-03-02

**Authors:** Pascal Amedro, Arthur Gavotto, Helena Huguet, Luc Souilla, Anne-Cecile Huby, Stefan Matecki, Anne Cadene, Gregoire De La Villeon, Marie Vincenti, Oscar Werner, Charlene Bredy, Kathleen Lavastre, Hamouda Abassi, Sarah Cohen, Sebastien Hascoet, Claire Dauphin, Aurelie Chalard, Yves Dulac, Nathalie Souletie, Helene Bouvaist, Stephanie Douchin, Matthias Lachaud, Caroline Ovaert, Camille Soulatges, Nicolas Combes, Jean-Benoit Thambo, Xavier Iriart, Fanny Bajolle, Damien Bonnet, Helene Ansquer, Jean-Guillaume Delpey, Laurence Cohen, Marie-Christine Picot, Sophie Guillaumont, the QUALIREHAB Study Group, Pascal Amedro, Arthur Gavotto, Helena Huguet, Luc Souilla, Anne-Cecile Huby, Johanna Calderon, Stefan Matecki, Anne Cadene, Gregoire De La Villeon, Marie Vincenti, Oscar Werner, D’Arcy Vandenberghe, Charlene Bredy, Kathleen Lavastre, Hamouda Abassi, Sarah Cohen, Sebastien Hascoet, Claire Dauphin, Aurelie Chalard, Yves Dulac, Nathalie Souletie, Philippe Acar, Helene Bouvaist, Stephanie Douchin, Matthias Lachaud, Caroline Ovaert, Camille Soulatges, Nicolas Combes, Jean-Benoit Thambo, Xavier Iriart, Emilie Testet, Fanny Bajolle, Antoine Legendre, Damien Bonnet, Helene Ansquer, Jean-Guillaume Delpey, Laurence Cohen, Victor Pommier, Remi Vincent, Frederique Sidney-Hetmaniak, Laurent Poirette, Sonia Corone, Cecile Rocca, Marianne Noirclerc, Oxana-Anca Neagu, Hervé Ngayap-Nemkam, Isaam Kammache, Clara Bourgarde, Jean-Marie Chevalier, Christelle Pons, Marie-Christine Picot, Sophie Guillaumont

**Affiliations:** Department of Fetal, Pediatric and Adult Congenital Cardiology, M3C National CHD Reference Centre, Bordeaux University Hospital, Haut-Leveque Hospital, Avenue de Magellan, 33604 Pessac Cedex, France; IHU Liryc, INSERM 1045, University of Bordeaux, Avenue du Haut-Leveque, 33600 Pessac, France; Pediatric and Congenital Cardiology Department, M3C Regional Reference CHD Centre, Montpellier University Hospital, Montpellier, France; PhyMedExp, INSERM, CNRS, University of Montpellier, Montpellier, France; Epidemiology and Clinical Research Department, University Hospital, University of Montpellier, Montpellier, France; PhyMedExp, INSERM, CNRS, University of Montpellier, Montpellier, France; Department of Fetal, Pediatric and Adult Congenital Cardiology, M3C National CHD Reference Centre, Bordeaux University Hospital, Haut-Leveque Hospital, Avenue de Magellan, 33604 Pessac Cedex, France; IHU Liryc, INSERM 1045, University of Bordeaux, Avenue du Haut-Leveque, 33600 Pessac, France; PhyMedExp, INSERM, CNRS, University of Montpellier, Montpellier, France; Epidemiology and Clinical Research Department, University Hospital, University of Montpellier, Montpellier, France; Pediatric and Congenital Cardiology Department, M3C Regional Reference CHD Centre, Montpellier University Hospital, Montpellier, France; Pediatric Cardiology and Rehabilitation Unit, St-Pierre Institute, Palavas-Les-Flots, France; Pediatric and Congenital Cardiology Department, M3C Regional Reference CHD Centre, Montpellier University Hospital, Montpellier, France; PhyMedExp, INSERM, CNRS, University of Montpellier, Montpellier, France; Pediatric Cardiology and Rehabilitation Unit, St-Pierre Institute, Palavas-Les-Flots, France; Pediatric and Congenital Cardiology Department, M3C Regional Reference CHD Centre, Montpellier University Hospital, Montpellier, France; Pediatric Cardiology and Rehabilitation Unit, St-Pierre Institute, Palavas-Les-Flots, France; Pediatric and Congenital Cardiology Department, M3C Regional Reference CHD Centre, Montpellier University Hospital, Montpellier, France; Fontfroide Cardiac Rehabilitation Center, 1800 rue de Saint-Priest, 34097 Montpellier, France; Pediatric and Congenital Cardiology Department, M3C Regional Reference CHD Centre, Montpellier University Hospital, Montpellier, France; Pediatric and Congenital Cardiology Department, M3C Regional Reference CHD Centre, Montpellier University Hospital, Montpellier, France; PhyMedExp, INSERM, CNRS, University of Montpellier, Montpellier, France; Pediatric and Congenital Cardiology Department, M3C National Reference CHD Centre, Marie-Lannelongue Hospital, Le Plessis-Robinson, France; Pediatric and Congenital Cardiology Department, M3C National Reference CHD Centre, Marie-Lannelongue Hospital, Le Plessis-Robinson, France; Pediatric and Congenital Cardiology Department, M3C Regional Reference CHD Centre, Clermont-Ferrand University Hospital, Clermont-Ferrand, France; Pediatric and Congenital Cardiology Department, M3C Regional Reference CHD Centre, Clermont-Ferrand University Hospital, Clermont-Ferrand, France; Pediatric and Congenital Cardiology Department, M3C Regional Reference CHD Centre, Toulouse University Hospital, Toulouse, France; Pediatric and Congenital Cardiology Department, M3C Regional Reference CHD Centre, Toulouse University Hospital, Toulouse, France; Pediatric and Congenital Cardiology Department, M3C Regional Reference CHD Centre, Grenoble University Hospital, Grenoble, France; Pediatric and Congenital Cardiology Department, M3C Regional Reference CHD Centre, Grenoble University Hospital, Grenoble, France; Pediatric and Congenital Cardiology Department, M3C Regional Reference CHD Centre, Grenoble University Hospital, Grenoble, France; Pediatric and Congenital Cardiology Department, M3C Regional Reference CHD Centre, APHM La Timone Hospital, Marseille, France; Pediatric and Congenital Cardiology Department, M3C Regional Reference CHD Centre, APHM La Timone Hospital, Marseille, France; Pediatric and Congenital Cardiology Department, Pasteur Clinic, Toulouse, France; Department of Fetal, Pediatric and Adult Congenital Cardiology, M3C National CHD Reference Centre, Bordeaux University Hospital, Haut-Leveque Hospital, Avenue de Magellan, 33604 Pessac Cedex, France; IHU Liryc, INSERM 1045, University of Bordeaux, Avenue du Haut-Leveque, 33600 Pessac, France; Department of Fetal, Pediatric and Adult Congenital Cardiology, M3C National CHD Reference Centre, Bordeaux University Hospital, Haut-Leveque Hospital, Avenue de Magellan, 33604 Pessac Cedex, France; Pediatric and Congenital Cardiology Department, M3C National Reference CHD Centre, APHP Necker Hospital, Paris, France; Pediatric and Congenital Cardiology Department, M3C National Reference CHD Centre, APHP Necker Hospital, Paris, France; Pediatric and Congenital Cardiology Department, Brest University Hospital, Brest, France; Pediatric and Congenital Cardiology Department, Brest University Hospital, Brest, France; Fetal, Pediatric and Congenital Private Practice, 8 rue du Conseil de l'Europe, 91300 Massy, France; Epidemiology and Clinical Research Department, University Hospital, University of Montpellier, Montpellier, France; Clinical Investigation Centre, INSERM-CIC 1411, University of Montpellier, Montpellier, France; Pediatric and Congenital Cardiology Department, M3C Regional Reference CHD Centre, Montpellier University Hospital, Montpellier, France; Pediatric Cardiology and Rehabilitation Unit, St-Pierre Institute, Palavas-Les-Flots, France

**Keywords:** Congenital heart defect, Physical activity, Physical fitness, Patient education, Quality of life, Exercise therapies

## Abstract

**Background and Aims:**

Cardiopulmonary fitness in congenital heart disease (CHD) decreases faster than in the general population resulting in impaired health-related quality of life (HRQoL). As the standard of care seems insufficient to encourage and maintain fitness, an early hybrid cardiac rehabilitation programme could improve HRQoL in CHD.

**Methods:**

The QUALIREHAB multicentre, randomized, controlled trial evaluated and implemented a 12-week centre- and home-based hybrid cardiac rehabilitation programme, including multidisciplinary care and physical activity sessions. Adolescent and young adult CHD patients with impaired cardiopulmonary fitness were randomly assigned to either the intervention (i.e. cardiac rehabilitation) or the standard of care. The primary outcome was the change in HRQoL from baseline to 12-month follow-up in an intention-to-treat analysis. The secondary outcomes were the change in cardiovascular parameters, cardiopulmonary fitness, and mental health.

**Results:**

The expected number of 142 patients was enroled in the study (mean age 17.4 ± 3.4 years, 52% female). Patients assigned to the intervention had a significant positive change in HRQoL total score [mean difference 3.8; 95% confidence interval (CI) 0.2; 7.3; *P* = .038; effect size 0.34], body mass index [mean difference −0.7 kg/m^2^ (95% CI −1.3; −0.1); *P* = .022; effect size 0.41], level of physical activity [mean difference 2.5 (95% CI 0.1; 5); *P* = .044; effect size 0.39], and disease knowledge [mean difference 2.7 (95% CI 0.8; 4.6); *P* = .007; effect size 0.51]. The per-protocol analysis confirmed these results with a higher magnitude of differences. Acceptability, safety, and short-time effect of the intervention were good to excellent.

**Conclusions:**

This early hybrid cardiac rehabilitation programme improved HRQoL, body mass index, physical activity, and disease knowledge, in youth with CHD, opening up the possibility for the QUALIREHAB programme to be rolled out to the adult population of CHD and non-congenital cardiac disease.

## Structured Graphical Abstract

**Structured Graphical Abstract:**
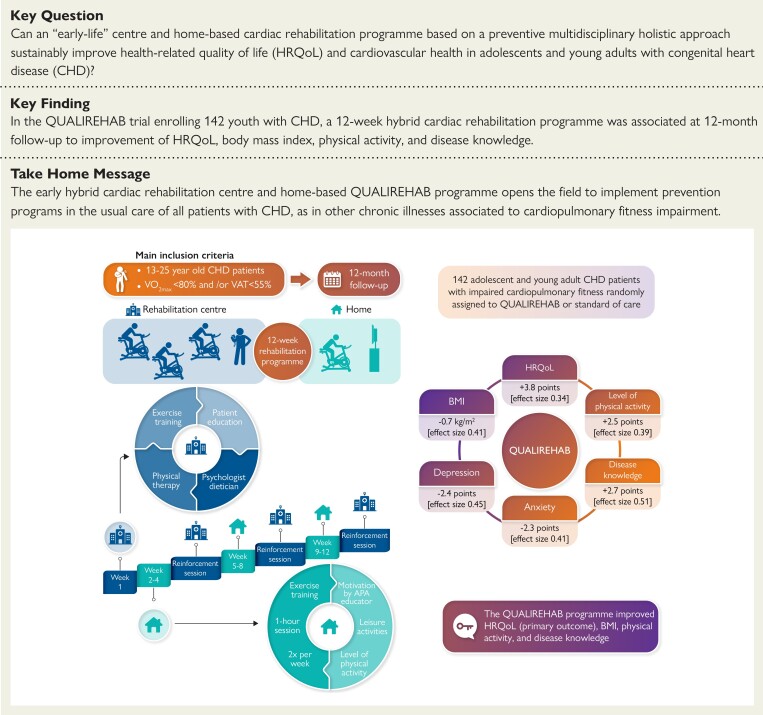
The long-term beneficial effect of the 12-week centre-based and home-based hybrid cardiac rehabilitation programme on adolescents and young adults with CHD was observed on HRQoL (primary outcome), BMI, physical activity, and disease knowledge, opening up the possibility for the QUALIREHAB programme to be rolled out to the adult population of CHD and non-congenital cardiac disease. BMI, body mass index; CHD, congenital heart disease; HRQoL, health-related quality of life.

**See the editorial comment for this article ‘Home-based fitness training: chicken soup for the ACHD soul?', by K.M. Shafer and A.M. Valente, https://doi.org/10.1093/eurheartj/ehae142.**

## Introduction

Significant advances in the management of paediatric chronic diseases have improved the overall prognosis and transferred mortality from childhood to adulthood, even in severe conditions.^1^ However, impaired cardiopulmonary fitness has been observed in various paediatric chronic diseases,^2–6^ cumulating to the underlying condition’s burden and participating in increased adult morbidity and cardiovascular risk.^7^ This ‘vicious circle’ of physical deconditioning is intertwined with physical inactivity and sedentary behaviours. Yet, most children with chronic diseases should be encouraged to follow the international guidelines on physical activity and sedentary behaviours and to engage in 60 min/day of moderate-to-vigorous–intensity aerobic physical activity across the week.^8^ Indeed, physical activity and exercise are associated with reduced chronic disease risks, improved mental health, and better quality of life.^9^

Impaired cardiopulmonary fitness is commonly marked by a decrease in maximum oxygen uptake (VO_2max_) and ventilatory anaerobic threshold (VAT), as observed by serial cardiopulmonary exercise testing (CPET).^2,3^ In the general adult population, a VO_2max_ decrease above 3.5 mL/kg/min is a predictor of all-cause mortality and cardiovascular events.^10,11^ In youth with repaired congenital heart disease (CHD), the negative impact of cardiopulmonary fitness impairment on quality of life has been established,^12^ despite tremendous advances in cardiac surgery and promotion of physical activity in the latest guidelines.^13,14^

Most young people with CHD are less physically active than their healthy peers,^14^ putting them at risk of becoming sedentary adults and acquiring latent chronic diseases.^15^ Barriers to physical activity in youth with CHD are multifactorial: reduced aerobic capacity inherent to the cardiac disease,^2^ impaired muscle function,^16^ impaired lung function,^17^ low self-concept and self-efficacy,^18^ parental attitude,^19^ home and health environment,^20^ or restriction recommendations by physicians.^21^ Ultimately, adults with CHD have a higher burden of adverse cardiovascular events relative to the general population and a high prevalence of smoking, obesity, hypertension, hyperlipidaemia, and diabetes mellitus.^22^

In adult heart failure, cardiac rehabilitation reduces cardiac morbidity and improves health-related quality of life (HRQoL).^23^ Cardiac rehabilitation is defined by the World Health Organization as ‘the coordinated sum of activity and interventions required to ensure the best possible physical, mental, and social conditions so that patients may, by their own efforts, preserve or resume their proper place in society and lead an active life’.^24^ Yet only a minority of patients receive appropriate exercise training, due to limited resources (lack of staff or health insurance) and difficulties encountered by patients (pressure from repeated hospital trips, reluctance to attend group sessions).^25^ These issues have contributed to the emergence of home-based cardiac rehabilitation programmes.^26^ However, in patients with CHD, home-based programmes have failed to demonstrate their effectiveness,^27^ probably due to a lack of personalization and multidisciplinary management, as it would be classically provided in a cardiac rehabilitation centre. In youth with CHD, the level of evidence for physical activity interventions remains limited.^9,27–30^ Cardiac rehabilitation involves not only exercise but also patient educational support. Transition education programmes dedicated to youth with CHD have addressed these specific needs,^31–33^ but their educational content has not been integrated into structured cardiac rehabilitation programmes. This gap in common practice may explain why the level of physical activity in youth with CHD seems to be related more to socio-behavioural factors than to the severity of heart disease.^34^

The current challenge is, therefore, to act as early as possible on the modifiable risk factors in the CHD population. An ‘early-life’ cardiac rehabilitation programme, designed for young people and based on a primary prevention multidisciplinary approach, is therefore justified.^35^ The efficacy of such a holistic programme should be ideally assessed by patient-reported outcomes,^36^ whose reliability has been largely demonstrated in the CHD population.^12,37,38^

In the QUALIREHAB multicentre randomized controlled trial, we aimed to assess the impact of a hybrid centre- and home-based cardiac rehabilitation programme on HRQoL, cardiovascular outcomes, cardiopulmonary fitness, and psychological outcomes, in adolescents and young adults with CHD.

## Methods

### Trial design and oversight

The QUALIREHAB trial is a prospective, multicentre, randomized, controlled, parallel-arm study, with a 12-month follow-up, and conducted in 12 CHD centres and 9 cardiac rehabilitation centres in France. Patients were screened in physicians’ offices, private clinics, tertiary care public institutions, and university hospitals. The trial design has been published previously.^39^

The study was supported by public and academic grants. The funders had no influence on the design or conduct of the trial and were not involved in data collection or analysis, in the writing of the manuscript, or in the decision to submit it for publication. The trial protocol was approved by a drawn National Ethics Committee and registered on a clinical trial registry. The study was conducted in compliance with the Good Clinical Practices protocol and Declaration of Helsinki principles. The authors assume responsibility for the accuracy and completeness of the data and analyses and the fidelity of the trial and this report to the protocol.

### Study population

Patients with a CHD, as defined by the international anatomical and clinical classification of CHD classification,^40^ and aged from 13 to 25 years old, were prospectively recruited in the clinical recruitment sites, during an outpatient visit. All types of CHD were eligible for the study, from simple forms such as ventricular septal defect to complex forms such as univentricular heart. Patients underwent a medical check-up including a cardiology consultation, an electrocardiogram, an echocardiography, and a CPET. Patients with impaired cardiopulmonary fitness, defined by a VO_2max_ < 80% and/or a VAT < 55% of predicted values, were eligible for the study.^2,39^ The main exclusion criteria were an absolute contraindication for CPET, a cardiac surgical procedure planned during the study, participation in a cardiac rehabilitation programme in the past 2 years, uncontrolled heart failure or arrhythmia, severe left ventricular outflow tract obstruction, pregnancy, or patient’s inability to understand and complete the HRQoL questionnaire. A complete list of the inclusion and exclusion criteria is provided in the Supplementary data online, *Appendix*. Patients were recruited consecutively in each centre, according to investigator availability, patient acceptance of rehabilitation constraints, and patient eligibility. Informed consent was obtained from all patients and their parents or legal guardians for minors.

### Trial procedures

Randomization was performed using a computer-generated assignment sequence, stratified by the clinical recruitment site. Participants were randomly allocated in a 1:1 ratio to either the intervention or the control group. The two groups were (i) the QUALIREHAB intervention group, e.g. patients participating in the cardiac rehabilitation programme, and (ii) the control group, e.g. patients undergoing a regular non-modified follow-up with no rehabilitation programme during the 12-month study period. The control group was not to receive any specific intervention for 1 year (new therapeutic education programme, new physical activity promotion programme, etc.), apart from routine care.

### Intervention

**Figure 1:**
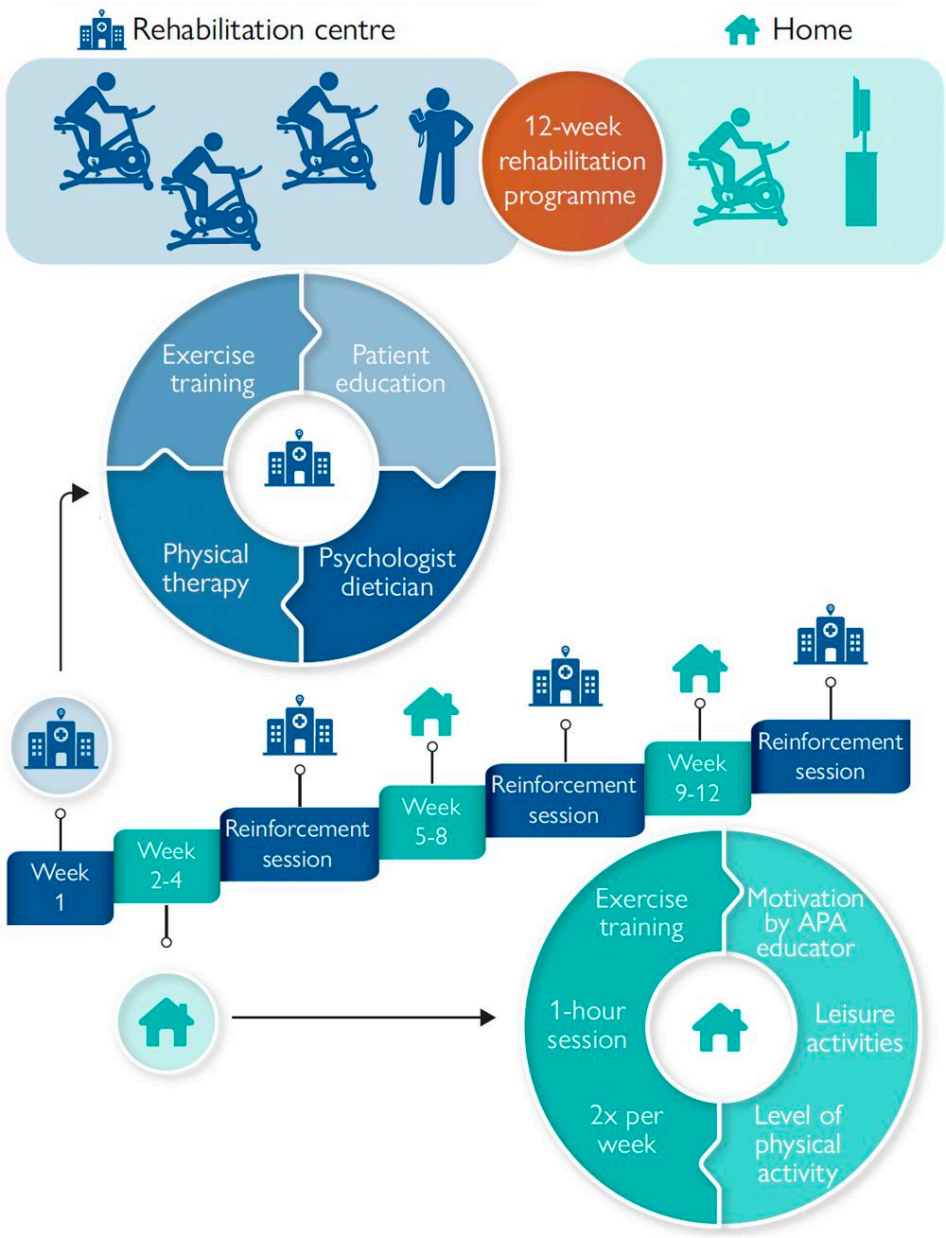
The QUALIREHAB intervention. The 12-week hybrid cardiac rehabilitation programme comprises a multidisciplinary initiation week (‘Week 1’) in a rehabilitation centre (hospital symbol), two sessions of 1-h exercise training per week for 11 weeks (Week 2 to Week 12) delivered by an adapted physical activity educator (house symbol), and 3 days of reinforcement session in the rehabilitation centre (hospital symbol). APA, adapted physical activity

The QUALIREHAB intervention was a 12-week ‘hybrid’ cardiac rehabilitation programme including a combined centre- and home-based training design. The same programme was delivered in all rehabilitation centres, with harmonization meetings prior to the start of the study. The home-based physical activity programme was carried out by a single nationwide health provider company (Stimulab©), selected after a national call for tenders, with no involvement in the scientific design of the study. The rehabilitation programme is summarized in *Figure 1* and detailed in the trial protocol.^39^

Subjects randomized to the QUALIREHAB intervention were offered an initiation week with their local rehabilitation centre (Day 1 to Day 5). First, the programme objectives and the patient’s educational needs were defined by the medical and nursing staff. The exercise training programme was a 1-h daily group session using a heart rate monitor watch (Garmin Forerunner® 25) and supervised by an adapted physical activity (APA) educator (one single APA educator per participant). The physical activity programme consisted of interval-training exercise at VAT’s heart rate level using a stationary bicycle.^41^ The patient’s motivation was also reinforced by various indoor (i.e. body weight resistance training, yoga, and dancing) and outdoor aerobic activities (i.e. fitness trail, paddleboard, and ball games). The multidisciplinary educational programme was delivered by psychologists, dieticians, and specialist nurses in both individual and group sessions, based on transition education guidelines for adolescents and young adults with CHD.^31,42,43^ Individual physical therapy sessions were delivered by a physiotherapist, including stretching, resistance, breathing, and thoracic extension exercises.

From Week 2 to Week 12, the home-based training programme involved two 1-h individual exercise sessions per week supervised by an APA educator, using the same protocol as in the rehabilitation centre and focusing on patient motivation reinforcement. One weekly session was held in person, at home, and the second one via videoconference. The homecare provider found an APA educator near the patient’s home, delivered the exercise equipment (stationary bike and Garmin Forerunner® 25 heart rate monitor watch), and provided access to the digital platform for the videoconference exercise sessions. Each patient was supervised by a single APA educator throughout the home programme, who was however different from the centre-based APA educator.

Three centre-based reinforcement day sessions were organized (one every 3 weeks), including a 1-h stationary bicycle interval-training exercise session supervised by an APA educator, an individual or group session of reinforced educational support with the specialist nurse, and an individual physical therapy session with a physiotherapist.

The end of the programme (Week 12) was organized at the rehabilitation centre, involving a CPET and a final multidisciplinary evaluation.

### Outcomes

Assessing patient-reported outcomes in cardiovascular clinical trials is relevant^36,39^ and fully justified in rehabilitation trials for youth with CHD, as HRQoL correlates with cardiopulmonary fitness in this population.^12^ Therefore, the primary outcome was the change in self-reported HRQoL score, using the PedsQL 4.0 questionnaire, between baseline and 12-month follow-up.^44^ The PedsQL generic HRQoL questionnaire has four multidimensional scales (physical functioning, emotional functioning, social functioning, and school functioning) and three summary scores ranging from 0 to 100 (total scale score, physical health summary, and psychosocial health summary, including emotional, social, and school functioning). Psychometric properties showed reliability, validity, and responsiveness to clinical change over time, including the French version of the PedsQL.^45,46^ In previous studies using various HRQoL instruments,^37,43,47,48^ our group also found a good sensitivity of the PedsQL generic questionnaire in the CHD population.^46,49–53^ We had initially considered several questionnaires for the QUALIREHAB trial, as other HRQoL instruments with good psychometric properties have been used in the paediatric and adult CHD populations. The KIDSCREEN-52 questionnaire is a 10-dimension validated generic paediatric HRQoL questionnaire,^54^ but, in our experience, the filling time is long,^37^ its construction provides little additional information to the PedsQL when both instruments have been used simultaneously, and it does not apply to the adult population.^46^ The SF-36 is a generic questionnaire validated in most countries,^55^ widely used in various chronic diseases, including CHD,^47^ with a short filling time, but it does not apply to the paediatric population. Specific HRQoL questionnaires, such as the PedsQL cardiac module, do not reflect the quality of life globally or in its main dimensions and preclude any comparison to the general population or other chronic diseases. Moreover, no HRQoL cardiac module has undergone linguistic and psychometric validation in the paediatric CHD population. Interestingly, the generic PedsQL instrument uses the same structure and scoring for all age groups, including adolescents and young adults: each item uses a 5-point Likert scale from 0 (never) to 4 (almost always), and items are reversed scored and linearly transformed to a 0–100 scale, higher scores indicating better HRQoL.^44^ Therefore, in the QUALIREHAB trial, adolescents and their parents completed the 13-to-17-year-old generic PedsQL self and proxy questionnaires. Young adults filled out the 18-to-25-year-old generic PedsQL self-reported questionnaire. All participants completed questionnaires before randomization.

Secondary outcomes were the change between baseline and 12-month follow-up in cardiovascular parameters [body mass index (BMI), blood pressure, and heart rate], physical health, mental health, and the level of disease knowledge. Physical health outcomes included CPET parameters [VO_2max_, VAT, ventilatory efficiency (VE/VCO_2_ slope), workload, and oxygen pulse]^2,56^ and self-reported level of physical activity.^57^ To be consistent with the pre-existing transition education programme dedicated to adolescents and young adults with CHD,^31,43^ we used the Ricci and Gagnon physical activity questionnaire, composed of nine items, with a total score ranging from 9 to 45 (9–17 = physical inactivity; 18–35 = moderate physical activity; and 36–45 = intensive physical activity).^58^ Exercise test procedures in all participating CPET laboratories were harmonized before the start of the study.^2^ To assess mental health, we identified commonly used and respected scores in the CHD population.^59^ Mental health outcomes evaluated the anxiety symptoms [State and Trait Anxiety Inventory (STAI) questionnaire for young adults, total score range = 20–80; STAI-Children questionnaire for adolescents, total score range = 20–60; higher scores indicating higher level of anxiety]^60^ and the depression symptoms [Beck’s Depression Inventory (BDI) questionnaire for young adults, total score range = 0–63; Child Depression Inventory (CDI) questionnaire for adolescents, total score range = 0–54; higher scores indicating higher level of depression].^61^ The level of disease knowledge was assessed by the Leuven Knowledge Questionnaire for Congenital Heart Disease (LKQCHD), a 27-item questionnaire covering five domains in which patients with CHD should be knowledgeable to be able to adopt adequate health behaviour (total score range = 0–100): the disease and its treatment; the prevention of complications; physical activities; sex and heredity; and contraception and pregnancy.^62,63^

Safety outcomes were assessed in both groups and prospectively collected from patient enrolment to the final visit (12-month follow-up). Serious and non-serious adverse events were analysed by the data safety and monitoring board members, blinded to the allocated group, to determine their relation to the intervention.

Acceptability and short-time effect of the cardiac rehabilitation programme were assessed in the intervention group. The acceptability of the cardiac rehabilitation programme to patients was determined by adherence to the programme, compliance to exercise sessions, and patient motivation. The adherence was estimated by the participation rates (number of sessions delivered vs. theoretical number of sessions), for the overall programme and for each component of the programme (i.e. centre-based initiation week, centre-based reinforcement sessions, and home-based sessions). Each one of the two APA educators in charge of the patient, at the centre and home, respectively, graded the patient’s compliance as poor, moderate, or good, based on the time spent per session in target heart rate zones. The patient’s motivation was subjectively graded by the APA educators as poor, moderate, or good, based on patient in-session engagement, compliance with instructions, in-session engagement, and autonomy regarding the heart rate monitor watch. The short-time effect of the rehabilitation programme was determined by the change in cardiovascular parameters (BMI, blood pressure, and heart rate) and cardiopulmonary fitness (VO_2max_, VAT, VE/VCO_2_ slope, workload, and oxygen pulse) between baseline and end-of-programme assessment at 12 weeks.

### Statistical analysis

The minimum clinically relevant difference was estimated from our previous HRQoL in the CHD population.^12,37,50^ The trial was designed to have 80% power to detect an absolute difference of 7 points (±13.5 points) in the change of the self-reported HRQoL total score with a two-sided alpha risk of 5% (nQuery software). At least 142 subjects were required to be conclusive, with potentially 20% of loss to follow-up and/or missing data on the primary outcome. All subjects enroled were included in the description of the population (baseline characteristics). An intention-to-treat (ITT) analysis was used. To comply with the ITT principle, when at least one of the four HRQoL multidimensional scores (physical, emotional, social, and school functioning) was missing at baseline or final assessment, multiple linear imputation was implemented using a fully conditional specification method. For better stability, the number of imputations was 20 in the imputation process, and all baseline characteristic data were used in the imputation model.^64^ A sensitivity analysis was performed on complete data (patients with complete baseline and final assessment). A per-protocol analysis was realized, including all randomized subjects with no important protocol deviation, and defined as follows: 18-month maximum delay between baseline and final primary outcome assessment, patients in the intervention group who have successfully completed the cardiac rehabilitation, with at least 80% of the sessions, and, for patients in the control group, a stable level of physical activity, i.e. less than an increase of +8 points, defined as clinically significant and also corresponding to the value of 1 SD. This last deviation was considered in order to limit the contamination bias due to the promotion of physical activity generated by the QUALIREHAB study during patient selection.

Baseline characteristics of the two groups were reported using means and SD for continuous variables and with frequencies and percentages for categorical variables. For each outcome, the change between baseline and 12-month follow-up was evaluated by a covariate analysis adjusted on the baseline value, age, sex, and allocated intervention group. To assess the centre effect and the influence of other confounding factors, a mixed effect model was used considering the clinical site both as a random and then as a fixed variable and adjusting on confounding factors. The effect size was estimated by the absolute difference of means and the value of Cohen’s *d* with their 95% confidence interval (CI). In the intervention group, changes between baseline and the end of the programme (Week 12) of the main clinical and CPET parameters were compared using paired Student’s *t*-test or Wilcoxon signed-rank test.

All tests were two sided with a statistical significance set as 0.05, and analyses were conducted using Statistical Analysis Systems version 7.13 (SAS Enterprise Guide).

## Results

### Patient characteristics

**Figure 2:**
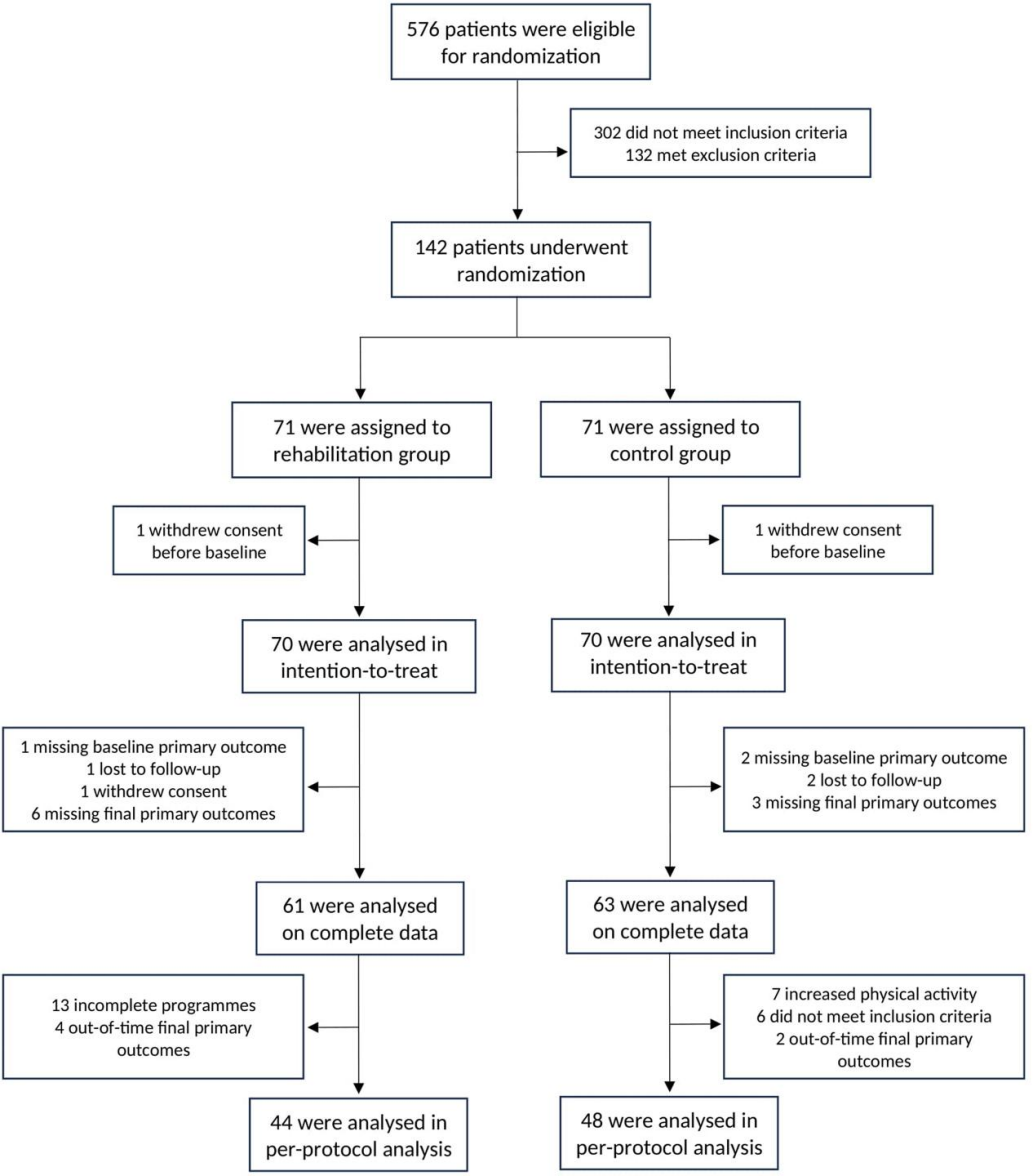
Flowchart. CONSORT diagram

Between July 2018 and March 2021, 142 patients (mean age 17.4 ± 3.4 years, 52% female) were enroled in the CHD centres, of which 70 were randomly assigned to the rehabilitation intervention group, and 70 were assigned to the control group (two patients withdrew their consent before baseline assessments; *Figure 2*). Overall, the two groups were balanced with respect to baseline characteristics (*Table 1*). The distribution of the number of cardiac surgeries seemed moderately inhomogeneous, but no statistically significant group difference was found. All types of CHD were represented in both groups, with most postnatal diagnoses (71%) and few genetic syndromes (6%). Most patients (83%) had undergone at least one cardiac surgical procedure and 44% at least one cardiac catheterization procedure. A mechanical valve or a pacemaker was present in 6% and 4%, respectively, of the patients, but none had an implantable cardioverter defibrillator. More than one-fourth (28%) were on cardiovascular medication. The mean systemic ventricle ejection fraction was normal (63 ± 9%). Overall, at baseline, enroled patients reported a moderate level of physical activity, a poor level of disease knowledge, no or minimal depression, and a moderate level of anxiety.

**Table 1:** Baseline population characteristics

	Rehabilitation group (*n* = 70)	Control group (*n* = 70)
Socio-demographic characteristics		
Age—years	17.1. ± 3.5	16.9 ± 3.1
Male sex—no. (%)	32 (46)	35 (50)
Level of education—no. (%)		
Middle school	33/69 (48)	29/70 (41)
High school	26/69 (38)	32/70 (46)
University	10/69 (14)	9/70 (13)
Living environment—no. (%)		
Living alone	5 (7)	6 (9)
Living as a couple	6 (9)	2 (3)
Living with parent(s)	53 (76)	57 (81)
Other	6 (9)	5 (7)
Cardiovascular outcomes		
Body mass index—kg/m^2^	21.8 ± 3.6	20.9 ± 4.0
Resting heart rate—b.p.m.	84.8 ± 18.6	82.9 ± 14.7
Resting arterial pressure		
Resting systolic arterial pressure—mmHg	119.5 ± 17.4	116.5 ± 15.4
Resting diastolic arterial pressure—mmHg	68.6 ± 12.3	69.0 ± 12.8
NYHA functional class—no. (%)		
I	35/63 (55)	26/48 (54)
II	27/63 (43)	20/48 (42)
III	1/63 (2)	2/48 (4)
Type of CHD—no. (%)		
Transposition of the great arteries	5/69 (7)	4/69 (6)
Atrial septal defect	9/69 (13)	5/69 (7)
Ventricular septal defect	12/69 (17)	7/69 (10)
Conotruncal defect	14/69 (21)	25/69 (37)
Aortic valve disease	5/69 (7)	5/69 (7)
Pulmonary valve disease	2/69 (3)	5/69 (7)
Coarctation of the aorta	8/69 (12)	5/69 (7)
Univentricular heart	9/69 (13)	5/69 (7)
Atrioventricular septal defect	2/69 (3)	5/69 (7)
Other	3/69 (4)	3/69 (5)
Cardiac surgeries—no. (%)		
0	13/69 (19)	10/70 (14)
1	35/69 (51)	25/70 (36)
≥2	21/69 (30)	35/70 (50)
Cardiac interventional catheterization—no. (%)		
0	46/69 (67)	40/68 (59)
1	15/69 (22)	16/68 (23)
≥2	8/69 (11)	12/68 (18)
Pacemaker—no. (%)	3 (4)	3 (4)
Mechanic valve—no. (%)	3 (4)	5 (7)
Patient under cardiac drugs—no. (%)	20/70 (29)	19/67 (28)
Cardiovascular comorbidity—no. (%)		
Thromboembolic event	1 (1)	1 (1)
Arrhythmia	12 (17)	5 (7)
Genetic anomalies—no. (%)	3 (4)	6/69 (9)
Other comorbidities—no. (%)	6 (9)	8 (11)
Echocardiography		
Systemic ventricular ejection fraction—%	61.9 ± 8.7	63.9 ± 9.8
HRQoL		
PedsQL self-reported scores		
Total score (range 0–100)	71.6 ± 15.2	68.3 ± 12.8
Physical functioning	73.5 ± 18.8	68.2 ± 15.7
Psychosocial health summary score	70.8 ± 15.4	68.2 ± 13.8
Emotional functioning	67.0 ± 22.9	62.6 ± 21.4
Social functioning	78.6 ± 18.9	76.0 ± 18.0
School functioning	66.6 ± 17.5	66.3 ± 16.7
PedsQL proxy-reported scores		
Total score (range 0–100)	67.9 ± 17.5	66.0 ± 13.2
Physical functioning	73.6 ± 20.9	69.4 ± 18.1
Psychosocial health summary score	65.2 ± 18.5	64.2 ± 14.7
Emotional functioning	62.1 ± 23.3	56.2 ± 22.6
Social functioning	73.0 ± 24.7	74.2 ± 17.8
School functioning	60.8 ± 19.2	62.1 ± 20.2
Disease knowledge		
LKQCHD, total score (range 0–100)	21.1 ± 6.4	21.2 ± 6.1
Physical health outcomes		
Level of physical activity, total score (range 9–45)	20.8 ± 7.0	19.8 ± 7.9
CPET parameters		
Spirometry		
FEV1—L	2.79 ± 0.85	2.76 ± 0.84
FVC—L	3.31 ± 1.10	3.29 ± 0.99
FEV1/FVC—%	85.01± 9.53	84.60 ± 11.08
Submaximal parameters		
VAT—mL/kg/min	17.6 ± 3.8	17.5 ± 4.4
Percent-predicted VAT—%	45.0 ± 10.7	44.0 ± 10.4
Heart rate at VAT—b.p.m.	128.6 ± 20.2	128.7 ± 22.9
Workload at VAT—W	73.5 ± 28.4	69.7 ± 25.2
VE/VCO_2_ slope	32.2 ± 6.9	32.5 ± 6.1
Maximal parameters		
VO_2max_—mL/kg/min	27.3 ± 5.9	27.1 ± 6.4
Percent-predicted VO_2max_—%	69.5 ± 10.6	67.9 ± 14.3
Maximum heart rate—b.p.m.	172.0 ± 22.4	168.7 ± 24.0
Maximum workload—W	127.7 ± 43.7	127.7 ± 34.7
Maximum oxygen pulse—mL/beat	9.48 ± 2.83	9.12 ± 2.47
Maximum RER	1.25 ± 0.16	1.22 ± 0.17
Mental health outcomes		
Anxiety symptoms in adolescents (STAI-C), total score (range 20–60)	31.9 ± 6.9	35.2 ± 8.6
Anxiety symptoms in young adults (STAI), total score (range 20–80)	44.2 ± 13.5	40.7 ± 9.1
Depression symptoms in adolescents (CDI), total score (range 0–54)	10.4 ± 6.3	12.7 ± 7.5
Depression symptoms in young adults (BDI), total score (range 0–63)	5.9 ± 5.9	4.8 ± 2.9

Plus–minus values are observed means ± SD.

BDI, Beck’s Depression Inventory (higher score indicates a higher level of depression symptoms); b.p.m., beats per minute; CDI, Child Depression Inventory (higher score indicates a higher level of depression symptoms); CHD, congenital heart disease; CPET, cardiopulmonary exercise testing; FEV1, forced expiratory volume in 1 s; FVC, forced vital capacity, HRQoL, health-related quality of life (higher score indicates a higher level of HRQoL); LKQCHD, Leuven Knowledge Questionnaire for Congenital Heart Disease (higher score indicates a higher level of disease knowledge); RER, respiratory exchange ratio; STAI, State and Trait Anxiety Inventory (higher score indicates a higher level of anxiety symptoms); STAI-C, State and Trait Anxiety Inventory for Children (higher score indicates a higher level of anxiety symptoms); VAT, ventilatory anaerobic threshold; VCO_2_, carbon dioxide production; VE, minute ventilation; VO_2max_, maximum oxygen uptake.

### Change in the primary outcome and health-related quality of life components

**Figure 3:**
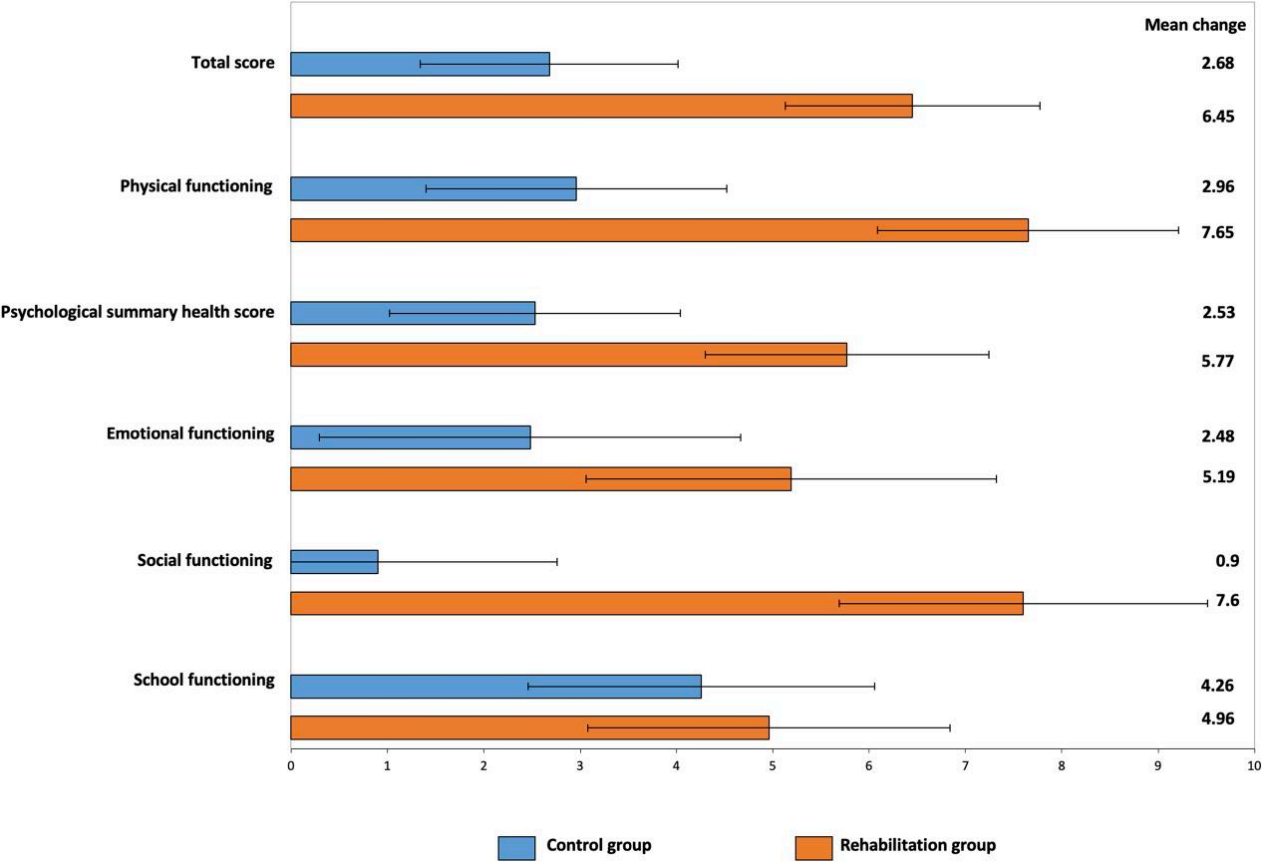
Change in health-related quality of life. This figure summarizes the change in primary outcome (self-reported total health-related quality of life score) and other self-reported health-related quality of life components between baseline and 12-month follow-up. For both intervention and control groups, bar plots represent the mean values of change adjusted on baseline health-related quality of life value, age, and sex. Error bars represent the standard error of the mean. The mean values of change are also indicated on the right of the graph

The ITT analysis with multiple imputation showed that the change in HRQoL self-reported total score between baseline and 12-month follow-up differed significantly between the intervention group and the control group, with a mean difference of 3.8 (95% CI 0.2; 7.3; *P* = .038, effect size 0.34), in favour of the cardiac rehabilitation group. This difference was also observed in self-reported physical health, with a mean difference of 4.7 (95% CI 0.4; 9.0, *P* = .033, effect size 0.36), self-reported social functioning, with a mean difference of 6.7 (95% CI 1.7; 11.7, *P* = .009, effect size 0.42), and proxy-reported HRQoL total score, with a mean difference of 7.4 (95% CI 0.1; 14.8, *P* = .048, effect size 0.38; *Table 2* and *Figure 3*; Supplementary data online, *Figure*). The mean delay between the end of rehabilitation and the final outcome assessment was 8 ± 3 months.

**Table 2:** Change in primary outcome and health-related quality of life components with intention-to-treat analysis using multiple imputations

	Rehabilitation group^a^	Control group^a^	Effect size [95% CI]^a^	Abs. diff. [95% CI]^a^	*P*-value^a^	Abs. diff. [95% CI]^b^	*P*-value^b^
Change in PedsQL self-reported scores	*n* = 70	*n* = 70					
Total score	6.45 ± 1.32	2.68 ± 1.34	0.34 [0.00; 0.67]	3.77 [0.21; 7.33]	.038	3.75 [0.12; 7.38]	.043
Physical functioning	7.65 ± 1.56	2.96 ± 1.56	0.36 [0.02; 0.70]	4.70 [0.37; 9.03]	.033	5.00 [0.54; 9.46]	.028
Psychological summary health score	5.77 ± 1.47	2.53 ± 1.51	0.26 [−0.08; 0.59]	3.24 [−0.72; 7.21]	.109	3.02 [−0.98; 7.02]	.139
Emotional functioning	5.19 ± 2.13	2.48 ± 2.19	0.15 [−0.18; 0.48]	2.71 [−3.25; 8.66]	.373	2.48 [−3.47; 8.44]	.413
Social functioning	7.60 ± 1.91	0.90 ± 1.86	0.42 [0.09; 0.76]	6.70 [1.70; 11.70]	.009	6.44 [1.40; 11.47]	.012
School functioning	4.96 ± 1.88	4.26 ± 1.80	0.05 [−0.29; 0.38]	0.70 [−4.35; 5.75]	.785	0.60 [−4.55; 5.76]	.819
Change in PedsQL proxy-reported scores	*n* = 48	*n* = 48					
Total score	8.30 ± 2.74	0.88 ± 2.86	0.38 [−0.03; 0.79]	7.42 [0.08; 14.77]	.048	7.39 [−0.28; 15.06]	.059
Physical functioning	6.47 ± 3.49	−2.95 ± 3.61	0.38 [−0.02; 0.79]	9.43 [−0.21; 19.08]	.055	9.91 [−0.05; 19.88]	.051
Psychological summary health score	9.43 ± 2.64	2.65 ± 2.65	0.37 [−0.04; 0.78]	6.78 [−0.06; 13.62]	.052	6.37 [−0.78; 13.51]	.081
Emotional functioning	11.49 ± 3.26	3.29 ± 3.45	0.35 [−0.05; 0.76]	8.20 [−0.90; 17.29]	.077	8.09 [−1.29; 17.47]	.091
Social functioning	6.37 ± 3.23	2.85 ± 3.10	0.16 [−0.24; 0.57]	3.52 [−4.59; 11.63]	.395	2.48 [−5.83; 10.80]	.558
School functioning	9.41 ± 3.19	2.06 ± 3.15	0.33 [−0.07; 0.74]	7.34 [−1.05; 15.73]	.086	7.09 [−1.61; 15.78]	.110

CI, confidence interval; Abs. diff., absolute difference.

^a^Plus–minus values are means adjusted on baseline HRQoL value, age, and sex ± standard error of the mean (SEM). The effect size represents the absolute value of Cohen’s *d*.

^b^Values adjusted on baseline HRQoL value, age, sex, number of cardiac surgeries, and clinical site.

The sensitivity analysis including only patients with complete data at baseline and final outcome assessments confirmed these findings for the primary outcome, with a mean difference of 3.7 (95% CI 0.1; 7.3, *P* = .046, effect size 0.36), and for the self-reported social functioning, with a mean difference of 6.4 (95% CI 1.4; 11.5, *P* = .013, effect size 0.46; see Supplementary data online, *Table S1*).

The per-protocol analysis also confirmed these results, with a higher magnitude of the difference for the primary outcome, with a mean difference of 5.2 (95% CI 0.8; 9.6, *P* = .021, effect size 0.50), the self-reported physical health, with a mean difference of 7.3 (95% CI 1.8; 12.5, *P* = .010, effect size 0.57), and the self-reported social functioning, with a mean difference of 7.3 (95% CI 1.3; 13.3, *P* = .017, effect size 0.52; see Supplementary data online, *Table S2*).

After adjustment on the number of cardiac surgeries and the clinical site, similar results were found with each of the two analyses (multiple imputation ITT and per-protocol analyses; *Table 2*; Supplementary data online, *Tables S1* and *S2*).

### Change in the secondary outcomes

In the analysis on patients with complete data, those assigned to the intervention had an improvement in BMI, with a mean difference of −0.7 kg/m^2^ (95% CI −1.3; −0.1, *P* = .022, effect size 0.41), self-reported level of physical activity, with a mean difference of 2.5 (95% CI 0.1; 5, *P* = .044, effect size 0.39), and the level of disease knowledge, with a mean difference of 2.7 (95% CI 0.8; 4.6, *P* = .007, effect size 0.51). In adolescents assigned to the intervention, we observed a trend for a decrease in anxiety symptoms (mean difference −2.33, 95% CI −4.88; 0.21, *P* = .07, effect size 0.41) and depression symptoms (mean difference −2.44, 95% CI −5.01; 0.13, *P* = .06, effect size 0.45). No significant change was observed in cardiopulmonary fitness parameters between baseline and 12-month follow-up.

The per-protocol analysis confirmed these results with a higher magnitude of the difference for the BMI, with a mean difference of −1.1 kg/m^2^ (95% CI −1.8; −0.4, *P* = .002, effect size 0.65), the self-reported level of physical activity, with a mean difference of 5.2 (95% CI 2.5; 7.8, *P* < .001, effect size 0.89), and the level of disease knowledge, with a mean difference of 3.7 (95% CI 1.3; 6.2, *P* = .003, effect size 0.69). A significant decrease in diastolic blood pressure at rest between baseline and 12-month follow-up was also observed in the per-protocol analysis, with a mean difference of −4.7 mmHg (95% CI −9.0; −0.5, *P* = .031, effect size 0.54; *Table 3*).

**Table 3:** Change in secondary outcomes in complete data and per-protocol analyses

	Complete data analysis (*N*_Rehab_ = 61/*N*_Control_ = 63)					Per-protocol analysis (*N*_Rehab_ = 46/*N*_Control_ = 44)				
	Rehabilitation group	Control group	Effect size [95% CI]	Abs. diff. [95% CI]	*P*-value	Rehabilitation group	Control group	Effect size [95% CI]	Abs. diff. [95% CI]	*P*-value
Cardiovascular outcomes										
Body mass index—kg/m^2^	0.35 ± 0.22	1.07 ± 0.22	0.41 [0.06; 0.75]	−0.72 [−1.34; −0.10]	.022	0.11 ± 0.25	1.19 ± 0.24	0.65 [0.23; 1.08]	−1.08 [−1.77; −0.39]	.002
Resting heart rate—b.p.m.	0.93 ± 1.57	−2.96 ± 1.59	0.32 [−0.05; 0.69]	3.88 [−0.55; 8.32]	.086	0.71 ± 1.97	−2.04 ± 2.00	0.21 [−0.22; 0.65]	2.75 [−2.84; 8.33]	.331
Resting systolic arterial pressure—mmHg	2.24 ± 2.69	1.76 ± 2.55	0.03 [−0.38; 0.43]	0.48 [−6.88; 7.85]	.897	2.95 ± 3.09	1.86 ± 2.74	0.06 [−0.42; 0.55]	1.09 [−7.20; 9.38]	.793
Resting diastolic arterial pressure—mmHg	−0.54 ± 1.53	1.75 ± 1.45	0.22 [−0.19; 0.63]	−2.28 [−6.48; 1.91]	.283	−4.17 ± 1.60	0.57 ± 1.42	0.54 [0.05; 1.03]	−4.74 [−9.02; −0.45]	.031
Level of disease knowledge	2.65 ± 0.68	−0.04 ± 0.70	0.51 [0.14; 0.88]	2.68 [0.76; 4.61]	.007	2.82 ± 0.84	−0.91 ± 0.86	0.69 [0.23; 1.14]	3.73 [1.32; 6.15]	.003
Level of physical activity	2.58 ± 0.88	0.06 ± 0.86	0.39 [0.01; 0.76]	2.52 [0.07; 4.97]	.044	3.44 ± 0.94	−1.72 ± 0.92	0.89 [0.42; 1.36]	5.16 [2.52; 7.81]	<.001
CPET parameters										
FEV1—L	0.24 ± 0.05	0.17 ± 0.06	0.19 [−0.23; 0.62]	0.08 [−0.1; 0.25]	.372	0.25 ± 0.08	0.11 ± 0.08	0.32 [−0.19; 0.83]	0.14 [−0.08; 0.36]	.214
FVC—L	0.27 ± 0.06	0.24 ± 0.06	0.07 [−0.37; 0.52]	0.03 [−0.15; 0.21]	.741	0.28 ± 0.08	0.17 ± 0.08	0.25 [−0.28; 0.79]	0.1 [−0.12; 0.33]	.350
FEV1/FVC—%	1.08 ± 1.31	0.14 ± 1.29	0.11 [−0.33; 0.56]	0.93 [−2.74; 4.61]	.614	0.17 ± 1.54	0.25 ± 1.52	0.01 [−0.52; 0.54]	−0.08 [−4.44; 4.28]	.971
VAT—mL/kg/min	0.94 ± 0.64	0.99 ± 0.63	0.01 [−0.35; 0.37]	−0.05 [−1.82; 1.73]	.959	0.43 ± 0.81	0.03 ± 0.77	0.08 [−0.35; 0.51]	0.40 [−1.83; 2.64]	.719
Percent-predicted VAT—%	2.37 ± 1.56	1.88 ± 1.53	0.04 [−0.32; 0.40]	0.48 [−3.85; 4.82]	.826	0.94 ± 1.96	−0.41 ± 1.87	0.11 [−0.32; 0.54]	1.35 [−4.03; 6.73]	.619
Heart rate at VAT—b.p.m.	3.06 ± 2.48	−0.15 ± 2.50	0.17 [−0.20; 0.54]	3.21 [−3.78; 10.2]	.365	2.39 ± 3.17	−0.42 ± 3.06	0.14 [−0.3; 0.58]	2.80 [−5.98; 11.58]	.527
Workload at VAT—W	10.86 ± 2.71	3.90 ± 2.64	0.34 [−0.03; 0.71]	6.96 [−0.56; 14.48]	.070	11.24 ± 3.45	2.31 ± 3.28	0.42 [−0.03; 0.87]	8.93 [−0.6; 18.45]	.066
VE/VCO_2_ slope	0.18 ± 0.77	1.47 ± 0.79	0.24 [−0.17; 0.64]	−1.29 [−3.49; 0.90]	.246	0.40 ± 0.94	0.99 ± 0.97	−0.10 [−0.58; 0.37]	0.58 [−2.12; 3.29]	.668
VO_2max_—mL/kg/min	1.02 ± 0.69	1.26 ± 0.69	0.04 [−0.31; 0.40]	−0.23 [−2.15; 1.7]	.815	0.92 ± 0.85	0.41 ± 0.84	0.09 [−0.33; 0.52]	0.51 [−1.88; 2.89]	.675
Percent-predicted VO_2max_—%	2.28 ± 1.73	2.55 ± 1.76	0.02 [−0.34; 0.37]	−0.27 [−5.16; 4.63]	.914	1.81 ± 2.17	0.33 ± 2.14	0.10 [−0.32; 0.53]	1.47 [−4.62; 7.57]	.631
Maximum heart rate—b.p.m.	−1.19 ± 2.32	−0.59 ± 2.35	0.03 [−0.32; 0.39]	−0.60 [−7.14; 5.93]	.855	−4.53 ± 2.50	−1.63 ± 2.45	−0.18 [−0.61; 0.25]	−2.90 [−9.87; 4.07]	.410
Maximum workload—W	12.43 ± 3.42	6.86 ± 3.49	0.21 [−0.15; 0.56]	5.57 [−4.11; 15.25]	.257	11.59 ± 3.82	5.66 ± 3.77	0.24 [−0.19; 0.67]	5.92 [−4.8; 16.65]	.275
Maximum oxygen pulse—mL/beat	0.77 ± 0.26	0.95 ± 0.26	0.10 [−0.28; 0.47]	−0.19 [−0.92; 0.55]	.615	0.91 ± 2.89	0.83 ± 0.29	0.04 [−0.4; 0.48]	0.08 [−0.73; 0.89]	.845
Mental health outcomes										
Anxiety symptoms in adolescents (STAI-C)	−2.38 ± 0.89	−0.05 ± 0.88	0.42 [−0.03; 0.87]	−2.33 [−4.88; 0.21]	.072	−0.33 ± 2.36	−0.53 ± 2.13	−0.37 [−0.89; 0.15]	−1.99 [−4.87; 0.89]	.172
Anxiety symptoms in young adults (STAI)	−0.64 ± 1.62	−1.57 ± 1.40	0.16 [−0.57; 0.89]	0.93 [−3.48; 5.34]	.668	−1.81 ± 1.01	0.18 ± 0.94	0.03 [−0.95; 1.01]	0.21 [−6.83; 7.25]	.951
Depression symptoms in adolescents (CDI)	−1.65 ± 0.93	0.79 ± 0.88	0.45 [−0.02; 0.93]	−2.44 [−5.01; 0.13]	.062	−0.70 ± 1.00	0.76 ± 0.91	−0.29 [−0.83; 0.25]	−1.45 [−4.28; 1.38]	.308
Depression symptoms in young adults (BDI)	−1.25 ± 0.81	−2.68 ± 0.74	0.44 [−0.25; 1.14]	1.43 [−0.84; 3.71]	.209	−3.13 ± 0.83	−1.70 ± 0.79	−0.57 [−1.55; 0.40]	−1.43 [−3.97; 1.11]	.247

Plus–minus values are means ± SD, adjusted on baseline value, age, and sex. The effect size represents the absolute value of Cohen’s *d*.

CI, confidence interval; Abs. diff., absolute difference.

After adjustment on the number of cardiac surgeries and the clinical site, similar results were found with each of the two analyses (complete data and per-protocol analyses; see Supplementary data online, *Table S3*).

### Acceptability, safety, and short-time effect of the intervention

The acceptability of the intervention was good, with the completion of >80% of the sessions for 81% of the patients. Participation rates were good for the centre-based initiation week (91%), the home-based physical activity sessions (88%), and the centre-based reinforcement sessions (77%). Participants’ motivation and compliance to exercise were good for 86% and 89% of patients, respectively, and all but one patient used the heart rate monitor watch appropriately (*Table 4*).

**Table 4:** Acceptability, safety, and short-time effect of the intervention

*n*		70	
Centre-based cardiac rehabilitation: first week + 3 reinforcement sessions			
Duration—days		7.6 ± 1	
Number of physical activity sessions		10.8 ± 1.8	
Number of therapeutic education sessions		5.9 ± 2.6	
Number of physiotherapy sessions		4.9 ± 2.1	
Number of psychotherapy sessions		1.4 ± 0.9	
Number of dietary sessions		2.2 ± 1.3	
Reinforcement sessions—no. (%)			
0		4 (6)	
1		3 (5)	
2		8 (12)	
3		49 (77)	
Home-based cardiac rehabilitation: Week 2 to Week 12			
Number of physical activity sessions		19.4 ± 5.8	
Acceptability			
Good adherence to full programme (>80% sessions completed)^a^—no. (%)		52/64 (81)	
Full participation to Week 1—no. (%)		64/70 (91)	
Good motivation to perform the exercise programme—no. (%)		48/56 (86)	
Good compliance to physical activity programme—no. (%)		50/56 (89)	
Appropriate use of heart rate monitor watch—no. (%)		54/55 (98)	
Safety			
Serious adverse events in the intervention group—no.		1^b^	
Suspension of rehabilitation after adverse event—no.		1^b^	
Adverse event related to rehabilitation—no.		0	
Adverse event related to underlying disease—no.		1^b^	
Hospitalization during intervention—no.		0	
**Effect of cardiac rehabilitation on the intervention group**	***n***	**Mean ± SD**	***P*-value**
Change in clinical outcomes between Week 1 and Week 12			
BMI—kg/m^2^	47	+0.13 ± 1.68	.497
Resting heart rate—b.p.m.	40	−7.90 ± 19.45	.014
Resting systolic arterial pressure—mmHg	34	+0.56 ± 14.21	.820
Resting diastolic arterial pressure—mmHg	34	−5.44 ± 13.08	.021
Change in CPET parameters between Week 1 and Week 12			
FEV1—L	30	+0.28 ± 0.36	<.001
FVC—L	28	+0.45 ± 0.65	.001
FEV1/FVC—%	28	−3.03 ± 11.15	.163
VAT—mL/kg/min	44	+4.70 ± 5.94	<.001
Percent-predicted VAT—%	43	+11.50 ± 14.55	<.001
Heart rate at VAT—b.p.m.	42	+12.40 ± 22.28	.001
Workload at VAT—W	36	+29.81 ± 30.48	<.001
VE/VCO_2_ slope	31	−1.36 ± 5.91	.210
VO_2max_—mL/kg/min	49	+3.02 ± 5.77	.001
Percent-predicted VO_2max_—%	45	+6.98 ± 12.81	<.001
Maximum heart rate—b.p.m.	51	+3.43 ± 15.29	.033
Maximum workload—W	49	+21.24 ± 34.32	<.001
Maximum oxygen pulse—mL/beat	34	+1.02 ± 1.98	.005

Values are means ± SD or *n* (%).

BMI, body mass index; FEV1, forced expiratory volume in 1 s; FVC, forced vital capacity, VAT, ventilatory anaerobic threshold; VCO_2_, carbon dioxide production; VE, minute ventilation; VO_2max_, maximum oxygen uptake.

^a^≥6 centre-based days and ≥17 home-based sessions.

^b^Pacemaker-mediated tachycardia.

The safety of the intervention was excellent, as no adverse event related to the rehabilitation programme was reported. Serious adverse events were observed in two patients in the control group (non-infectious febrile inflammatory syndrome with favourable outcome, suspicion of transient ischaemic attack during baseline CPET with normal neuroimaging and favourable outcome) and in one patient in the intervention group (pacemaker-mediated tachycardia that occurred 5 months after the end of rehabilitation).

When focusing on the immediate effect of rehabilitation in the intervention group (i.e. short-time effect), cardiovascular parameters and cardiopulmonary fitness significantly improved between baseline and the end of the rehabilitation programme (Week 12) for heart rate at rest (−7.9 b.p.m.), diastolic blood pressure at rest (−5.4 mmHg), VO_2max_ (+3.0 mL/kg/min), VAT (+4.7 mL/kg/min), and for most other CPET parameters (*Table 4*).

### Assessment of contamination bias

The control group received no specific intervention for 1 year, apart from routine care. The level of physical activity in the control group did not differ significantly between baseline and 12-month follow-up, with a mean difference of 0.1 ± 0.9 points (95% CI −1.7; 1.8). An increase in the level of physical activity superior to 1 SD (8 points) was observed for seven patients in the control group, who were excluded from the per-protocol analysis.

## Discussion

The QUALIREHAB national multicentre randomized controlled trial investigated the effect of a hybrid cardiac rehabilitation programme on mid-term outcomes in youth with CHD-related impaired cardiopulmonary fitness. This trial demonstrated that a 12-week centre- and home-based cardiac rehabilitation intervention improved HRQoL in adolescents and young adults with CHD. Furthermore, with excellent safety and good acceptability, the intervention was effective in improving cardiovascular outcomes, disease knowledge, and the level of physical activity (*Structured Graphical Abstract*).

Previous randomized controlled trials in young patients with CHD failed to clearly demonstrate the efficacy of physical activity interventions on HRQoL or physical capacity, probably due to insufficient exercise intensity or supervision, poor patient adherence, or paucity of patient education.^27–30^ Similarly in adults with CHD, a meta-analysis of three randomized controlled trials found a non-significant improvement in HRQoL (standardized mean difference 0.76) with very low certainty of evidence.^28^ In children and adolescents with CHD, recent literature reported that exercise training was safe, but its effectiveness had low-to-moderate certainty of evidence, probably because previous studies mainly enroled patients with only one or two types of CHD, delivered only home programmes, or assessed short-term effects immediately after rehabilitation.^30^ From a non-selective trial on all types of CHD, and using a hybrid programme, the QUALIREHAB study demonstrated a long-lasting effect of rehabilitation, after 1 year of follow-up, on both HRQoL and level of physical activity.^28^

The holistic approach of the QUALIREHAB programme may explain why intervention-related benefits seen in the trial exceeded previously reported values for the minimal clinically important difference.^28^ Indeed, the mean HRQoL group difference in ITT analysis (+3.8) was close to the reported minimal clinically important difference for the PedsQL instrument (+4.3)^44^ and even exceeded this limit in the per-protocol analysis (+5.2). This holistic nature of the intervention may have also contributed to the positive effects on multidimensional levels of HRQoL (physical health, social functioning, and proxy reports).

The significant mean increase of 3 mL/kg/min in VO_2max_ observed after the rehabilitation programme represents an important finding. Indeed, in the general population, a VO_2max_ increase of 3.5 mL/kg/min (i.e. one metabolic equivalent of task) is associated with an 11% decrease in all-cause mortality,^11^ and, in CHD, cardiopulmonary fitness has been associated with future prognosis.^65^ In the 15 randomized controlled trials pooled in the meta-analysis by Williams *et al.*,^28^ physical activity interventions were equally effective regardless of the type of CHD included, with a mean overall increase of 1.9 mL/kg/min in VO_2max_. Thus, the ‘hybrid’ nature of QUALIREHAB, combining a multidisciplinary centre-based component and a supervised home-based exercise training component, seems tailored to the population of young patients with CHD affected by cardiopulmonary fitness impairment.^3^ This hybrid model probably also facilitated the acceptability of the intervention, in particular by limiting the number of days in the hospital (eight in total) and maximizing the duration of exercise training at home (11 weeks). Supervision provided a safety net and comforting reassurance that a regular visit to the gym may not be able to offer.

The important place of patient education in this programme certainly helped to maintain a positive effect of the intervention at 12-month follow-up. Indeed, improvements were observed in all domains commonly targeted by patient education in cardiac rehabilitation: control of cardiovascular risk factors, by lowering blood pressure and BMI^66^; maintenance of physical activity beyond the intervention^34^; and promotion of patient autonomy by improving disease knowledge.^33^ The educational component of the rehabilitation programme was based on a pre-existing transition programme dedicated to adolescents and young adults with CHD.^31,43^ These results are supported by the STEPSTONES randomized controlled trial, which recently demonstrated the effectiveness of a structured patient education transition programme^32^ in increasing empowerment in adolescents with CHD.^33^

Apart from patient education, behavioural and psychological aspects probably played a role in maintaining physical activity after the end of the 3-month rehabilitation programme, thus contributing to the long-term beneficial effect. Classically, in the general youth population, as in young people with CHD population, physical activity is associated with a lower risk of psychiatric symptoms and a better quality of life.^67,68^ Unfortunately, despite a trend for a decrease in anxiety and depression symptoms, the impact of the intervention on mental health outcomes did not reach statistical significance in this trial, possibly due to a lack of mental health-focused intervention. Therefore, considering the prevalence of neurodevelopmental disorders in youth with CHD,^69^ our group will evaluate in the upcoming QUALINEURO-REHAB trial, a neuro-cardiac programme merging neurocognitive intervention with a cardiac rehabilitation programme in this population (https://clinicaltrials.gov/study/NCT05670132).

This study has several limitations. Other parameters could have been measured such as behavioural (smoking, alcohol), dietary, or biological (cholesterol, glycated haemoglobin, and blood sugar) changes during the rehabilitation programme. Some of the parameters measured by the APA educator are subjective (motivation and engagement) and may vary according to the participant’s CHD (compliance). The heterogeneity and size of CHD subgroups limited the analysis of the impact of the programme on patients with rare and severe conditions, such as univentricular hearts, which will be further studied. The average cost for the home-based part of the programme was 1500€, including the APA educator’s interventions and the equipment loan (stationary bike and heart rate monitor watch), but a comprehensive medico-economic analysis of the hybrid QUALIREHAB programme as a whole would be interesting.

## Conclusions

In conclusion, the QUALIREHAB centre and home-based cardiac rehabilitation programme improved HRQoL, BMI, blood pressure, physical activity, and disease knowledge, in adolescents and young adults with CHD. To induce behaviour change, generate sustainable cardiopulmonary fitness increase, and, ultimately, reduce adult cardiovascular morbidity, future programmes could combine high-intensity exercise, exercise progress monitoring, various patterns of training (i.e. exergame), and, most importantly, post-rehabilitation support. This early hybrid programme opens the field to implement prevention programmes in the usual care of all patients with CHD. QUALIREHAB could also be evaluated in other chronic illnesses in young people with impaired cardiopulmonary fitness.

## Supplementary Material

ehae085_Supplementary_Data

## Data Availability

The data underlying this article will be shared upon reasonable request to the corresponding author.

## Pre-registered Clinical Trial Number

The pre-registered clinical trial number on Clinicaltrials.gov is NCT03690518.

## Appendix

**Author information—QUALIREHAB Study Group**

Pascal Amedro (Bordeaux University Hospital, France), Arthur Gavotto (Montpellier University Hospital, France), Helena Huguet (Montpellier University Hospital, France), Luc Souilla (University of Montpellier, France), Anne-Cecile Huby (Bordeaux University Hospital, France), Johanna Calderon (University of Montpellier), Stefan Matecki (Montpellier University Hospital, France), Anne Cadene (Montpellier University Hospital, France), Gregoire De La Villeon (Montpellier University Hospital, France), Marie Vincenti (Montpellier University Hospital, France), Oscar Werner (Montpellier University Hospital, France), D’Arcy Vandenberghe (Montpellier University Hospital, France), Charlene Bredy (Montpellier University Hospital, France), Kathleen Lavastre (Montpellier University Hospital, France), Hamouda Abassi (Montpellier University Hospital, France), Sarah Cohen (Marie-Lannelongue Hospital, France), Sebastien Hascoet (Marie-Lannelongue Hospital, France), Claire Dauphin (Clermont-Ferrand University Hospital, France), Aurelie Chalard (Clermont-Ferrand University Hospital, France), Yves Dulac (Toulouse University Hospital, France), Nathalie Souletie (Toulouse University Hospital, France), Philippe Acar (Toulouse University Hospital, France), Helene Bouvaist (Grenoble University Hospital, France), Stephanie Douchin (Grenoble University Hospital, France), Matthias Lachaud (Grenoble University Hospital, France), Caroline Ovaert (La Timone Marseille University Hospital, France), Camille Soulatges (La Timone Marseille University Hospital, France), Nicolas Combes (Pasteur Clinic, Toulouse, France), Jean-Benoit Thambo (Bordeaux University Hospital, France), Xavier Iriart (Bordeaux University Hospital, France), Emilie Testet (Bordeaux University Hospital, France), Fanny Bajolle (APHP Necker Hospital, Paris, France), Antoine Legendre (APHP Necker Hospital, Paris, France), Damien Bonnet (APHP Necker Hospital, Paris, France), Helene Ansquer (Brest University Hospital, France), Jean-Guillaume Delpey (Brest University Hospital, France), Laurence Cohen (Massy Fetal, Pediatric and Congenital Private Practice, France), Victor Pommier (Montpellier University Hospital, France), Remi Vincent (Montpellier University Hospital, France), Frederique Sidney-Hetmaniak (Fontfroide Cardiac Rehabilitation Clinic, France), Laurent Poirette (Louis Berard Cardiac Rehabilitation Centre, France), Sonia Corone (Bligny Cardiac Rehabilitation Center, France), Cecile Rocca (Grenoble University Hospital, France), Marianne Noirclerc (Grenoble University Hospital, France), Oxana-Anca Neagu (Beaumont-de-Lomagne Cardiac Rehabilitation Centre, France), Hervé Ngayap-Nemkam (Chateau des Cotes Cardiac Rehabilitation Centre, France), Isaam Kammache (Chateau des Cotes Cardiac Rehabilitation Centre, France), Clara Bourgarde (Chateau des Cotes Cardiac Rehabilitation Centre, France), Jean-Marie Chevalier (Avicenne Cardiac Rehabilitation Centre, France), Christelle Pons (Ty Yann Cardiac Rehabilitation Centre, France), Marie-Christine Picot (Montpellier University Hospital, France), and Sophie Guillaumont (Montpellier University Hospital, France).
