# Supplementary material for: Early hybrid cardiac rehabilitation in congenital heart disease: the QUALIREHAB trial
Source: Eur Heart J. 2024 Mar 2;45(16):1458–73. doi: 10.1093/eurheartj/ehae085 (PMC11032713; doi:10.1093/eurheartj/ehae085)
Supplement: ehae085_Supplementary_Data [file ehae085_supplementary_data.docx]

**Supplementary Appendix**

| **Inclusion criteria** |
| --- |
| - Male or female aged 13 to 25 years old. |
| - Patients with a CHD, as defined by the international ACC-CHD classification. |
| - CPET performed within the last 3 months with VO2_max_ < 80% of predicted VO2_max_ or VAT < 55% of predicted VO2_max_. |
| - Written informed consent for adult patients, or legal guardians for teenagers, and formal assent for teenagers. |
| **Exclusion criteria** |
| - Absolute contraindications for CPET: fever, uncontrolled asthma, respiratory failure, acute myocarditis or pericarditis, uncontrolled arrhythmias causing symptoms or hemodynamic compromise, uncontrolled heart failure, acute pulmonary embolus or pulmonary infarction, and children with mental impairment leading to inability to cooperate. |
| - Cardiac surgery planned during the study. |
| - Patient who has undertaken cardiac rehabilitation within the last 24 months. |
| - Uncontrolled arrhythmia. - Advanced atrioventricular block. |
| - Uncontrolled heart failure (NYHA IV). - Uncontrolled hypertension. - Acute myocarditis and pericarditis. - Symptomatic aortic stenosis. - Severe hypertrophic obstructive cardiomyopathy. - Acute systemic illness. - Recent (< 3 months) intracardiac thrombus, embolism, or thrombophlebitis. |
| - Pregnancy. |
| - Severe musculoskeletal abnormalities. |
| - Patients who are unable to understand the study information or unable to complete study procedures. - Patients with a severe intellectual disability that does not allow the completion of the quality-of-life questionnaire. - Patients who are in a long-term care institution or who are unwilling or unable to travel to research assessments or accommodate home visits. - Patients considered being unable to participate in the study for any other reason (for example, psychiatric disorder, dementia, life-threatening comorbidity, etc.). - Patients participating in concurrent interventional research which may overburden the patient or confound data collection. |

**Supplementary Table S1. Change in the primary outcome and HRQoL components in complete data analysis***

|  | **Rehabilitation**  **Group** | **Control**  **Group** | **Effect size**  **[95% CI]** | **Abs. diff.**  **[95% CI]** |  | **P-value** |
| --- | --- | --- | --- | --- | --- | --- |
| **Change in PedsQL self-reported scores** | *N=61* | *N=63* |  |  |  |  |
| Total score | 6.96 ± 1.29 | 3.72 ± 1.27 | 0.36 [0.01 ; 0.72] | 3.68 [0.06 ; 7.31] |  | 0.046 |
| Physical functioning | 7.85 ± 1.62 | 3.65 ± 1.59 | 0.33 [-0.02 ; 0.69] | 4.21 [-0.33 ; 8.74] |  | 0.069 |
| Psychosocial summary health score | 6.34 ± 1.45 | 3.00 ± 1.42 | 0.30 [-0.06 ; 0.66] | 3.34 [-0.69 ; 7.38] |  | 0.103 |
| Emotional functioning | 5.77 ± 2.17 | 3.00 ± 2.12 | 0.16 [-0.19 ; 0.52] | 2.77 [-3.25 ; 8.79] |  | 0.364 |
| Social functioning | 8.35 ± 1.82 | 1.92 ± 1.77 | 0.46 [0.10 ; 0.82] | 6.43 [1.38 ; 11.48] |  | 0.013 |
| School functioning | 5.29 ± 1.89 | 3.72 ± 1.86 | 0.11 [-0.25 ; 0.46] | 1.57 [-3.70 ; 6.83] |  | 0.557 |
| **Change in PedsQL proxy-reported scores** | *N=37* | *N=36* |  |  |  |  |
| Total score | 6.72 ± 2.66 | 2.87 ± 2.69 | 0.24 [-0.23 ; 0.71] | 3.85 [-3.80 ; 11.51] |  | 0.319 |
| Physical functioning | 5.30 ± 3.50 | -0.20 ± 3.58 | 0.26 [-0.21 ; 0.73] | 5.50 [-4.62 ; 15.63] |  | 0.282 |
| Psychosocial summary health score | 8.24 ± 255 | 3.64 ± 2.59 | 0.30 [-0.17 ; 0.78] | 4.60 [-2.74 ; 11.94] |  | 0.215 |
| Emotional functioning | 10.88 ± 3.16 | 5.16 ± 3.20 | 0.30 [-0.17 ; 0.78] | 5.73 [-3.44 ; 14.90] |  | 0.217 |
| Social functioning | 5.81 ± 3.05 | 1.96 ± 3.00 | 0.21 [-0.26 ; 0.69] | 3.85 [-4.75 ; 12.46] |  | 0.374 |
| School functioning | 7.82 ± 3.24 | 3.41 ± 3.29 | 0.23 [-0.25 ; 0.70] | 4.41 [-4.88 ; 13.70] |  | 0.347 |

* Plus–minus values are means adjusted on baseline HRQoL value, age, and sex ± standard error of the mean (SEM). The effect size represents the absolute value of Cohen’s d. CI, confidence interval. Abs. diff., absolute difference.

**Supplementary Table S2. Change in the primary outcome and HRQoL components in the per-protocol analysis**

|  | **Rehabilitation**  **Group *** | **Control**  **Group *** | **Effect size**  **[95% CI] *** | **Abs. diff.**  **[95% CI] *** |  | **P-value *** | **Abs. diff.**  **[95% CI] †** | **P-value †** |
| --- | --- | --- | --- | --- | --- | --- | --- | --- |
| **Change in PedsQL self-reported scores** | *N=44* | *N=48* |  |  |  |  |  |  |
| Total score | 7.11 ± 1.56 | 1.89 ± 1.50 | 0.50 [0.08 ; 0.92] | 5.22 [0.81 ; 9.63] |  | 0.021 | 5.22 [0.81 ; 9.63] | 0.021 |
| Physical functioning | 8.96 ± 1.91 | 1.81 ± 1.82 | 0.57 [0.14 ; 0.99] | 7.15 [1.77 ; 12.53] |  | 0.010 | 7.15 [1.77 ; 12.53] | 0.010 |
| Psychosocial summary health score | 5.76 ± 1.75 | 1.79 ± 1.65 | 0.35 [-0.07 ; 0.77] | 3.97 [-0.91 ; 8.85] |  | 0.109 | 3.97 [-0.90 ; 8.85] | 0.109 |
| Emotional functioning | 4.60 ± 2.57 | 0.01 ± 2.43 | 0.27 [-0.14 ; 0.69] | 4.60 [-2.57 ; 11.76] |  | 0.206 | 4.60 [-2.57 ; 11.76] | 0.206 |
| Social functioning | 7.89 ± 2.16 | 0.59 ± 2.04 | 0.52 [0.09 ; 0.94] | 7.29 [1.31 ; 13.27] |  | 0.017 | 7.54 [1.62 ; 13.46] | 0.013 |
| School functioning | 5.66 ± 2.36 | 4.15 ± 2.23 | 0.10 [-0.32 ; 0.51] | 1.50 [-5.00 ; 8.01] |  | 0.647 | 1.50 [-5.00 ; 8.01] | 0.647 |
| **Change in PedsQL proxy-reported scores** | *N=29* | *N=31* |  |  |  |  |  |  |
| Total score | 6.92 ± 2.87 | 3.01 ± 2.78 | 0.25 [-0.26 ; 0.77] | 3.91 [-4.23 ; 12.06] |  | 0.340 | 3.91 [-4.23 ; 12.06] | 0.340 |
| Physical functioning | 6.45 ± 3.84 | -0.64 ± 3.71 | 0.34 [-0.18 ; 0.86] | 7.09 [-3.80 ; 17.99] |  | 0.197 | 7.09 [-3.80 ; 17.99] | 0.197 |
| Psychosocial summary health score | 7.76 ± 2.77 | 4.55 ± 2.63 | 0.22 [-0.30 ; 0.74] | 3.20 [-4.56 ; 10.97] |  | 0.412 | 3.20 [-4.56 ; 10.97] | 0.412 |
| Emotional functioning | 10.68 ± 3.36 | 5.95 ± 3.21 | 0.27 [-0.26 ; 0.79] | 4.74 [-4.90 ; 14.38] |  | 0.329 | 4.74 [-4.90 ; 14.38] | 0.329 |
| Social functioning | 6.28 ± 3.51 | 2.59 ± 3.27 | 0.20 [-0.32 ; 0.73] | 3.69 [-6.00 ; 13.39] |  | 0.449 | 3.69 [-6.00 ; 13.39] | 0.449 |
| School functioning | 5.82 ± 3.61 | 4.53 ± 3.43 | 0..07 [-0.45 ; 0.59] | 1.29 [-8.80 ; 11.37] |  | 0.799 | 1.29 [-8.80 ; 11.37] | 0.799 |

* Plus–minus values are means adjusted on baseline HRQoL value, age, and sex ± standard error of the mean (SEM). The effect size represent the absolute value of Cohen’s d. † Values adjusted on baseline value, age, sex, number of cardiac surgeries, and clinical site. CI, confidence interval. Abs. diff., absolute difference.

**Supplementary Table S3. Change in secondary outcomes in complete data and per-protocol analyses***

|  | **Analysis with complete data**  ***(N _Rehab_ = 61 / N _Control_ = 63)*** | |  | **Per protocol analysis**  ***(N _Rehab_ = 46 / N _Control_ = 44)*** | |
| --- | --- | --- | --- | --- | --- |
|  | **Abs. diff.**  **[95% CI]** | **P-value** |  | **Abs. diff.**  **[95% CI]** | **P-value** |
| **Cardiovascular outcomes** |  |  |  |  |  |
| Body mass index – Kg/m^2^ | -0.73 [-1.35 ; -0.11] | 0.022 |  | -0.99 [-1.66 ; -0.31] | 0.005 |
| Resting heart rate – bpm | 4.53 [0.07 ; 8.99] | 0.047 |  | 3.07 [-2.49 ; 8.63] | 0.275 |
| Resting systolic arterial pressure – mmHg | 0.87 [-6.75 ; 8.49] | 0.821 |  | 0.89 [-7.60 ; 9.39] | 0.834 |
| Resting diastolic arterial pressure – mmHg | -2.25 [-6.56 ; 2.06] | 0.303 |  | -5.11 [-9.44 ; -0.77] | 0.022 |
| **Level of disease knowledge** | 2.84 [0.87 ; 4.81] | 0.005 |  | 3.91 [1.44 ; 6.38] | 0.002 |
| **Level of physical activity** | 2.61 [0.16 ; 5.05] | 0.037 |  | 5.20 [2.55 ; 7.84] | <0.001 |
| **CPET parameters** |  |  |  |  |  |
| FEV1 – L | 0.10 [-0.08 ; 0.28] | 0.261 |  | 0.14 [-0.09 ; 0.36] | 0.219 |
| FVC – L | 0.06 [-0.13 ; 0.24] | 0.543 |  | 0.11 [-0.11 ; 0.33] | 0.332 |
| FEV1/FVC – % | 1.12 [-2.61 ; 4.84] | 0.551 |  | 0.26 [-4.16 ; 4.68] | 0.905 |
| VAT – mL/Kg/min | -0.36 [-2.09 ; 1.37] | 0.679 |  | 0.06 [-2.08 ; 2.20] | 0.957 |
| Percent-predicted VAT – % | -0.38 [-4.56 ; 3.80] | 0.858 |  | 0.38 [-4.73 ; 5.49] | 0.883 |
| Heart rate at VAT – bpm | 2.90 [-4.06 ; 9.86] | 0.411 |  | 2.74 [-5.94 ; 11.42] | 0.532 |
| Workload at VAT – Watts | 5.72 [-1.78 ; 13.23] | 0.134 |  | 8.45 [-0.84 ; 17.74] | 0.074 |
| VE/VCO_2_ slope | -1.16 [-3.38 ; 1.06] | 0.300 |  | -0.68 [-3.36 ; 1.99] | 0.612 |
| VO_2max_ – mL/Kg/min | -0.43 [-2.35 ; 1.49] | 0.657 |  | 0.16 [-2.10 ; 2.42] | 0.890 |
| Percent-predicted VO_2max_ – % | -0.95 [-5.74 ; 3.85] | 0.696 |  | 0.29 [-5.40 ; 5.98] | 0.919 |
| Maximum heart rate – bpm | -0.55 [-7.29 ; 6.18] | 0.871 |  | -2.86 [-9.98 ; 4.26] | 0.427 |
| Maximum workload – Watts | 6.48 [-2.90 ; 15.86] | 0.174 |  | 6.12 [-4.45 ; 16.68] | 0.252 |
| Maximum oxygen pulse – mL/beat | -0.24 [-0.93 ; 0.46] | 0.499 |  | 0.01 [-0.77 ; 0.79] | 0.984 |
| **Mental Health Outcomes** |  |  |  |  |  |
| Anxiety symptoms in adolescents (STAI-C) | -2.22 [-4.84 ; 0.40] | 0.096 |  | -2.16 [-5.11 ; 0.80] | 0.149 |
| Anxiety symptoms in young adults (STAI) | 1.91 [-2.83 ; 6.65] | 0.413 |  | -0.59 [-8.34 ; 7.16] | 0.869 |
| Depression symptoms in adolescents (CDI) | -2.28 [-4.97 ; 0.41] | 0.096 |  | -0.99 [-3.95 ; 1.97] | 0.503 |
| Depression symptoms in young adults (BDI) | 0.90 [-1.49 ; 3.30] | 0.445 |  | -0.95 [-3.45 ; 1.56] | 0.427 |

* Values adjusted on baseline value, age, sex, number of cardiac surgeries, and clinical site. CI, confidence interval. Abs. diff., absolute difference.

**Supplementary Figure. HRQoL baseline and final scores in rehabilitation and control groups**


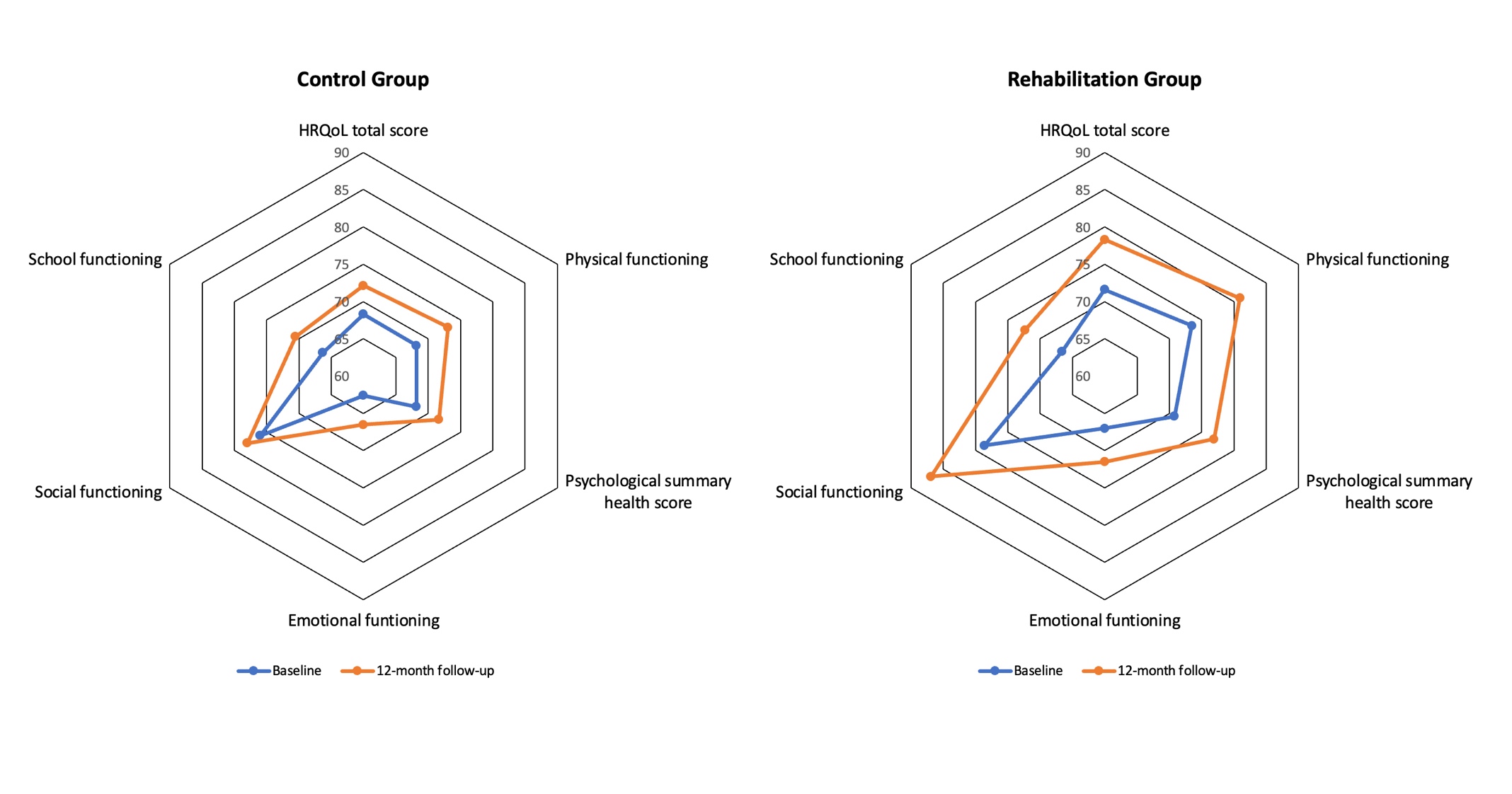


Legend: Baseline (blue colour) and final 12-month follow-up (orange colour) self-reported HRQoL mean total and component scores with the PedsQL in the intervention group (cardiac rehabilitation) and the control group.
